# Scenario-Based Simulation for Pain Assessment and Control in a Postanesthesia Care Unit

**DOI:** 10.1590/0034-7167-2024-0421

**Published:** 2025-10-03

**Authors:** Elizabete Cristina de Lira Santiago, Giulia Moreira Dias, Ana Clara de Sousa Timote, Joanlise Marco de Leon Andrade, Michelle Zampieri Ipolito, Mani Indiana Funez

**Affiliations:** I Universidade de Brasilia, School of Health Sciences and Technology. Brasilia, Federal District, Brazil; II Universidade de Brasilia, Department of Statistics. Brasilia, Federal District, Brazil

**Keywords:** Patient Simulation, Patient Safety, Pain Management, Postoperative Period, Nursing, Simulação de Paciente, Segurança do Paciente, Manejo da Dor, Período Pós-Operatório, Enfermagem, Simulación de Paciente, Seguridad del Paciente, Manejo del Dolor, Periodo Posoperatorio, Enfermería

## Abstract

**Objective:**

to construct, validate, and test a realistic simulation scenario for pain control in a Postanesthesia Care Unit.

**Methods:**

a methodological, cross-sectional, quantitative study, developed in an undergraduate nursing course in three phases: scenario construction; content validity; scenario testing. It was approved by the Research Ethics Committee of the School of Ceilândia, *Universidade de Brasília*. The Item-Level Content Validity Index (I-CVI), Scale-Level Content Validity Index/Average (S-CVI/Ave) and Scale-Level Content Validity Index/Universal Agreement (S-CVI/UA) were analyzed. The exact binomial test was used to assess whether the proportion of agreement among judges was equal to or greater than 80%.

**Results:**

a high-complexity and fidelity scenario was developed, containing the stages of pre-briefing, briefing, simulation, and debriefing. All the scenario assessment items by experts reached a CVI of 80%, a S-CVI/UA of 85% and a S-CVI/Ave of 0.98%.

**Conclusion:**

the proposed scenario was successfully constructed, validated and tested.

## RESUMO

**Objetivo:**

construir, validar e testar cenário de simulação realista para controle da dor em Unidade de Recuperação Pós-Anestésica.

**Métodos:**

estudo metodológico, transversal, quantitativo, desenvolvido em curso de graduação em enfermagem em três fases: construção do cenário; validade de conteúdo; teste do cenário. O estudo foi aprovado pelo Comitê de Ética em Pesquisa da Faculdade de Ceilândia, Universidade de Brasília. Foram analisados os *Item-Level Content Validity Index* (I-CVI), *Scale-Level Content Validity Index/Average* (S-CVI/Ave) e *Scale-Level Content Validity Index/Universal Agreement* (S-CVI/UA). O teste binomial exato foi utilizado para avaliar se a proporção de concordância entre os juízes foi igual ou superior a 80%.

**Resultados:**

foi desenvolvido cenário de alta complexidade e fidelidade, contendo as etapas de pré-*briefing, briefing*, simulação e *debriefing*. Todos os itens de avaliação do cenário por especialistas atingiram IVC de 80%, S-CVI/UA de 85% e S-CVI/Ave de 0,98%.

**Conclusão:**

o cenário proposto foi construído, validado e testado com sucesso.

## RESUMEN

**Objetivo:**

construir, validar y probar un escenario de simulación realista para el control del dolor en una Unidad de Cuidados Postanestésicos.

**Métodos:**

estudio metodológico, transversal y cuantitativo, desarrollado en un curso de enfermería de pregrado en tres fases: construcción del escenario; validez de contenido; prueba del escenario. Fue aprobado por el Comité de Ética en Investigación de la Facultad de Ceilândia, *Universidade de Brasilia*. Se analizaron el *Item-Level Content Validity Index* (I-CVI), *Scale-Level Content Validity Index/Average* (S-CVI/Ave) y *Scale-Level Content Validity Index/Universal Agreement* (S-CVI/UA). Se utilizó la prueba binomial exacta para evaluar si el porcentaje de concordancia entre los jueces era igual o superior al 80%.

**Resultados:**

se desarrolló un escenario de alta complejidad y fidelidad, que comprende las etapas de *pre-briefing, briefing*, simulación y *debriefing*. Todos los elementos de la evaluación del escenario por parte de los expertos alcanzaron un IVC del 80%, un S-CVI/UA del 85% y un S-IVC/Ave del 0,98%.

**Conclusión:**

el escenario propuesto se construyó, validó y probó con éxito.

## INTRODUCTION

A Postanesthesia Care Unit (PACU) is where patients are received (pediatric, adult, older adults) who undergo anesthetic-surgical procedures, from all specialties^(1)^. In most countries around the world, the nursing team is responsible for patient care, with an anesthetist and surgeon available on call^(1)^. Care in the PACU aims to ensure anesthetic recovery with a focus on preventing anesthetic and surgical complications. Pain is a post-operative complication and a relevant outcome during post-operative recovery. Its assessment and control by the nursing team in PACU is critical for the quality of care^(1)^. Kiekkas *et al*. (2021)^(2)^ investigated missed nursing care in the PACU, and found a prevalence of 78.1%. Regardless of its cause, it is important to highlight that one of the three most reported was “care associated with pain” ^(2)^. Thus, post-operative pain control in the PACU is a challenge for the nursing team. Furthermore, pain control is an outcome for hospital discharge^(3)^. With the implementation of Enhanced Recovery After Surgery (ERAS) programs, ensuring that pain is controlled at hospital discharge is essential and can impact the flow of patients in the PACU. Likewise, reducing the time for pain control and length of stay in the PACU are indicators that impact greater patient satisfaction^(4)^.

Simulation is a strategy that has potential as an educational intervention for nurses’ pain management. The benefits of scenario-based simulation have been seen in various areas of nursing education, including physical examination, psychiatric, critical care, and medical-surgical nursing^(5-9)^. Its versatility is an advantage because it allows for the creation of scenarios with applicability in different areas.

The International Nursing Association for Clinical Simulation and Learning and Best Evidence Medical Education^(10)^ recommends that, for the development of realistic health simulation scenarios, some steps should be followed to ensure quality. Among them, scenario validity stands out, which has been shown to have an impact on participants’ learning. Validated scenarios are more effective in promoting undergraduate nursing students’ knowledge when compared to non-validated scenarios^(11)^.

Given the importance of adequate pain control in PACU and the relevance of simulation strategies in health, the importance of developing realistic simulation scenarios in this area is evident, whether for undergraduate students or for training teams in institutions providing health services. To the best of our knowledge, we have not found a validated scenario in international studies for realistic simulation in the PACU focused on nursing pain management.

## OBJECTIVE

To construct, validate, and test a realistic simulation scenario for pain control in a postanesthesia recovery room.

## METHODS

### Ethical aspects

The study was approved by the Research Ethics Committee of the *Universidade de Brasília, Faculdade de Ciências e Tecnologias em Saúde* (supplementary material), and was conducted in accordance with Resolution 466/2012 of the Brazilian National Health Council: Guidelines and Regulatory Norms for Research Involving Human Subjects (Brazil, National Health Council, 2012).

### Study design, period, and location

This is a methodological study with a cross-sectional design and a quantitative approach. The simulation-based research extensions for the Strengthening the Reporting of Observational Studies in Epidemiology (STROBE) Statement recommendations were followed. The study was developed in three phases: scenario construction; content validity by experts; and scenario testing by experts and undergraduate students. Scenario construction took place from July to December 2022. Scenario content validity took place online from January to July 2023. Scenario testing was conducted on September 9, 2023 at the Skills and Care Simulation Laboratory of the Nursing Undergraduate Program, *Universidade de Brasília, Faculdade de Ciências e Tecnologias em Saúde*.

### Population or sample; inclusion and exclusion criteria

Eleven professionals/experts and four undergraduate nursing students were selected by convenience sampling. For the selection process, experts’ curricula were obtained from the *Lattes* Platform^(12)^. Professional activities in the area of interest of the study were analyzed using the following criteria: specialization, master’s degree, doctorate, article publication, and a minimum of one year of clinical practice^(13)^. Students were required to be enrolled in the undergraduate nursing program, having been approved in the course subjects that qualified them to take part in the proposed scenario (fundamental cycle subjects such as semiology and nursing semiotechnics, and theoretical and practical subjects in the area of perioperative nursing), and aged ≥ 18 years.

### Study protocol

Phase 1 (scenario construction): this phase began with a literature review on acute pain control in the PACU by the nursing team. The scenario objectives were established based on Bloom’s Taxonomy, respecting the hierarchical levels of learning, using the cognitive, affective, and psychomotor domains^(14,15)^. The objectives guided the choice of a high-fidelity and complex scenario using SimMan^®^ Essential mannequin (Laerdal, Brazil). The simulation scenario proposal included the phases of pre-briefing, briefing, simulation, and debriefing^(10,16,17)^.

Phase 2 (scenario validity): experts were contacted virtually and invited to participate in the research; all accepted. Subsequently, an Informed Consent Form (supplementary material), a sociodemographic questionnaire, a scenario proposal, and a scenario assessment instrument were sent to each one. There were three experts who did not return the documentation, so eight experts participated in the subsequent phases of the study. The scenario was assessed by experts who assigned scores from “1” to “4” for 20 items that deal with general aspects such as plausibility and realism, objectives, promotion of problem-solving, debriefing, among others^(18)^. Validity was carried out by the Content Validity Index (CVI), which utilizes a Likert-type scale, where the maximum score was “4” and the minimum “1”, considering an 80% agreement among experts^(19,20)^. The score “1” corresponds to assessment of an item as “inadequate”, the score 2, to “needs to be reformulated”, the score 3, to “adequate with the possibility of revision”, and the score 4, to “adequate” ^(19)^.

Phase 3 (scenario testing): this phase took place in person and included eight experts, four undergraduate nursing students, the research team, and the laboratory technical team. An invitation to students was made virtually or in person. Upon acceptance, an Informed Consent Form (supplementary material) was signed, and guidance was given for continuation of the research. Scenario testing aimed to verify the feasibility for executing the proposal that was built and validated in the previous stages^(19,20)^. The scenario was tested in two sessions, which took place on the same day, with two students participating in each session. Experts assessed the same items proposed in the scenario validity stage.

### Analysis of results, and statistics

Each item from the scenario assessment was analyzed individually by calculating the Item-Level Content Validity Index (I-CVI), which corresponds to the sum of response values “3” and “4” for each expert, divided by the number of responses for that item^(19)^. The scenario assessment scale was analyzed by calculating the Scale-Level Content Validity Index/Average (sum of all CVIs calculated separately divided by the number of items) (S-CVI/Ave) and the Scale-Level Content Validity Index/Universal Agreement (total number of items considered relevant - answers “3” and “4” - divided by the number of items) (S-CVI/UA)^(19,20)^. The exact binomial test was used to test whether the proportion of agreement among judges for each questionnaire item was equal to or greater than 80%. Results with p-values greater than or equal to 0.05 were considered non-significant, or, in this context, that there was no disagreement among judges. Data were analyzed using R software version 4.3.2^(21)^.

## RESULTS

The simulation scenario was validated by eight nurses, seven of whom were female and one male. The average age of experts was 33 years (ranging from 26 to 46 years old). The average time since graduation was nine years (ranging from 3 to 18 years), and the mean time of experience in the area of interest was eight years (ranging from 3 to 18 years). Experts had experience in undergraduate and graduate nursing education, continuous education, and care in the following areas: (03) perioperative nursing and adult health; (03) realistic simulation; and (02) perioperative nursing and pain.

Validity occurred in two stages, allowing for necessary adjustments (Table 1). In the first stage, content validity analysis showed that the majority of items reached a CVI of 80%. The items related to debriefing did not reach the established CVI (I-CVI 0.38; p=0.01), which impacted the percentage of items with unanimous agreement on the scale (S-CVI/UA; 75). Despite this, the average of the scale’s CVI was satisfactory (S-CVI/Ave; 0.89). Items that received a score of “1” or “2” were removed or reformulated according to experts’ suggestions, and items with a score of “3” were reviewed accordingly. On this occasion, experts made observations about the lack of information on students’ level of prior knowledge and skills, and the absence of such information provided in briefing and debriefing. The revised proposal was sent to experts for the second stage of validity, in which all items reached a CVI of 80%. Furthermore, the indices obtained from scale analysis were also adequate (S-CVI/UA; 85 and S-CVI/Ave; 0.98). Accordingly, the content proposed by the scenario was considered validated.

**Table 1:** Validity and testing of a scenario scenario-based simulation for pain assessment and control in a Postanesthesia Care Unit. Brasília, Federal District, Brazil, 2024

Item assessed	Scenario validity CVI		Scenario testing CVI	
	1^st^ stage	2^nd^ stage	Session 1	Session 2
*Plausibility of clinical case*	1.00	1.00	1.00	1.00
*Realism*	1.00	1.00	1.00	1.00
*Adherence to available scientific evidence*	1.00	1.00	1.00	1.00
*Complexity in relation to students’ level of knowledge and skills*	0.88	1.00	1.00	1.00
*Case summary*	1.00	1.00	1.00	1.00
*Simulation objectives given to students*	1.00	0.88	1.00	1.00
*Information given to students before simulation*	0.75	1.00	1.00	1.00
*Data given to students during simulation*	1.00	1.00	1.00	1.00
*Support given to students during simulation*	1.00	1.00	1.00	1.00
*Learning objective*	1.00	0.88	1.00	1.00
*Promotion of critical thinking*	1.00	1.00	1.00	1.00
*Promotion of the ability to prioritize nursing assessments and interventions*	1.00	1.00	1.00	1.00
*Promotion of autonomous problem-solving*	1.00	1.00	1.00	1.00
*Simulator type*	1.00	1.00	1.00	1.00
*Simulator parameters*	1.00	1.00	1.00	1.00
*Simulated environment*	1.00	1.00	1.00	1.00
*Available materials and equipment*	1.00	1.00	1.00	1.00
*Debriefing questions*	0.38^a^	0.88	0.75	0.75
*Reflections and analysis of actions in debriefing*	0.38^a^	1.00	0.75	0.75
*Synthesis and feedback to students in debriefing*	0.38^a^	1.00	0.88	0.88
*S-CVI-AVE*	0.89	0.98	0.97	0.97
*S-CVI-UA*	75	85		

Note: CVI - Content Validity Index, exact binomial test; S-CVI-AVE - Scale-Level Content Validity Index/Universal Agreement; S-CVI-UA - Scale-Level Content Validity Index/Universal Agreement;

a Statistically significant result from the exact binomial test (p-value < 0.05).

**Figure 1:**
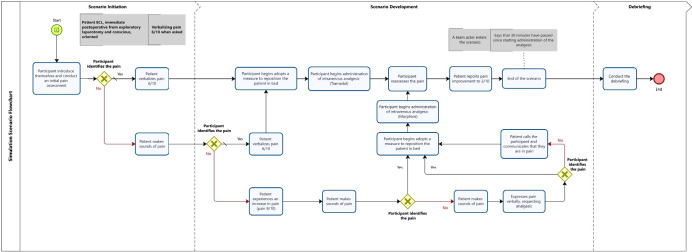
Simulation scenario flowchart

The scenario consisted of a patient in the PACU with rapid clinical deterioration due to acute pain. It was expected that students would demonstrate competence in assessing acute pain, clinical reasoning, and the development of nursing care for pain management (Chart 1 - short version, Figure 1). For the scenario testing session, a flowchart was constructed containing students’ possible actions and the responses triggered in the patient (Figure 1). They anticipated the expected outcome and complications of clinical picture. Moreover, the flowchart guided a script for the research team, which included the patient’s responses in dialogues, their occurrences (e.g., moans, sighs), complications and their signs and symptoms (e.g., in case of worsening pain, increased heart rate and blood pressure), organized according to the times of the scenario.

**Chart 1:** Description of a scenario-based simulation for pain assessment and control in a Postanesthesia Care Unit (short version). Brasília, Federal District, Brazil, 2024

Proposed simulation scenario: nursing care for patients with pain in a Postanesthesia Care Unit.
**Target audience:** undergraduate nursing students.
**Participant’s prior knowledge:** patient safety, drugs indications/contraindications/mechanism of action, nursing care in pain control, immediate postoperative nursing care, and Postanesthesia Care Unit.
**Learning objectives:** **General:** recognize and make decisions for proper pain control in a Postanesthesia Care Unit. **Specific:** assess pain, identifying its origin, location, duration, and intensity (pain numeric rating scale); utilize pharmacological and non-pharmacological strategies or pain control.
**Scenario time duration:** 5 minutes for pre-briefing, 15 minutes for simulation, and 30 minutes for debriefing, totaling 50 minutes.
**Simulators and equipment:** high-fidelity mannequin SimMan^®^ Essential (Laerdal, Brazil).
**Clinical case/situation:** **Immediate postoperative/Postanesthesia Care Unit:** patient ECL was admitted to a Postanesthesia Care Unit in immediate postoperative care following exploratory laparotomy, under the residual effects of general anesthesia.
**Debriefing:** gather participants and encourage reflective learning through self-assessment. At this time, participants will be guided to engage in reflective learning following the phases of reaction, analysis, and synthesis.

Pre-briefing began 15 days before the scenario test (Chart 1) and aimed to provide guidance that would assist in the execution of the scenario and clinical judgment, increasing confidence in dealing with problems, improving cognitive and affective skills as well as support for psychomotor skills. Students received written material on the topic and were asked to conduct individualized studies. The material covered nursing care in the PACU, nursing care for immediate postoperative complications, emphasizing pain assessment and control. Ten days before the test, an online and synchronous theoretical class was given, expository-dialogue style, where students had the opportunity to clarify queries about the material provided. Two days before the test, a practical class was held in the laboratory where materials and equipment were made available to students for reviewing nursing techniques such as medication administration, among others. There was also a recognition of the environment, which is an important step as it increases the participant’s safety, confidence, and autonomy.

On the day of the test, briefing involved information about the scenario, the patient’s pre-operative and intraoperative clinical picture (Chart 1). A presentation was made in the laboratory of the physical structure of the practical station, with devices, available equipment, and the high-fidelity mannequin. At this time, students could ask questions about any item they were unfamiliar with. The scenario began with the report of the patient’s clinical-surgical picture, support materials (medical records, assessment scales, medication prescription) and support team (two members of the research team). The first test session lasted 08 minutes and 30 seconds, and the second session lasted 13 minutes and 58 seconds. A debriefing was conducted after each test session and was structured in three phases: reaction; analysis; and synthesis (Chart 1).

At the end of the two test sessions, a qualitative assessment was made with four students, eight experts, and the research team. Everyone sat in a circle, and time was given for them to freely comment on their impressions, assessments, and even emotions regarding the execution of the scenario. This moment was led by the research team leader, and it was possible to identify that the proposal was feasible for practical execution, that the general time availability was adequate, that the information contained in prescription and medical records was sufficient, and, furthermore, that the realism of the scenario was highlighted by both participants and experts. Taking into account experts’ suggestion to give students more time during debriefing, the final version of the scenario was corrected. Students also highlighted the positive contributions that the experience brought to their training.

The analysis of validity indices of items showed that, for both test sessions, all items achieved a CVI of 80%. The indices obtained from scale analysis were also adequate (S-CVI/UA; 85 and S-CVI/Ave; 0.97, both sessions) (Table 1). Experts made comments about the scale items related to debriefing, such as use more time for questioning, promote greater reflection, and synthesis. There were suggestions regarding scenario organization, material arrangement, and the need to highlight some information about the patient’s clinical status. After analyzing the data, the last adjustments, changes in the scenario organization and planning processes were made. These data resulted in the final version of the proposal for a realistic simulation scenario for pain control in the PACU, as per Chart 1.

## DISCUSSION

In this study, the construction, validity, and testing of a high-fidelity realistic simulation scenario was carried out for pain control by the nursing team in the PACU.

“Translating pain knowledge to practice” is a challenge that was proposed by the International Association for the Study of Pain in its 2022 global campaign^(22)^ and continues to this day. A systematic review with meta-analysis that investigated types of education in pain management recommends that educational interventions that incorporate interactivity are necessary to improve health students’ skills^(23)^. To meet these demands, scenario-based simulation can be a useful strategy. Accordingly, the scenario construction in this study and established learning objectives were aligned with important concepts involving postoperative pain treatment by the nursing team. Pain is a post-operative complication and a relevant outcome during post-operative recovery. Pain impacts vital signs, contributing to the destabilization of the clinical condition, which causes extreme discomfort to patients and delays discharge from the PACU. Its assessment and control by the nursing team in the PACU are critical for patient satisfaction, quality of care, and successful hospital discharge. Gynecological surgery has a high risk of pain chronicity^(24)^, and the case constructed in this study additionally involved an oncological condition and major surgery, which are factors associated with higher levels of postoperative pain^(25)^. In this case, the recommendation for pain control involves multimodal analgesia (non-steroidal anti-inflammatory, gabapentinoids, intravenous local anesthetics, anxiolytics), which includes the use of opioids intravenously^(25)^. It is also necessary to respect the WHO analgesic ladder principles, combining the drugs indicated according to the level of pain presented by patients, considering the Numeric Pain Rating Scale and medical prescription^(26)^. Nonpharmacological pain management is widely recommended since pain has several dimensions^(27)^. Moreover, it should be part of pain management education programs for surgical nurses^(28)^. In this sense, including this content for undergraduate students becomes quite relevant. Patient positioning is a physical method with potential to establish comfort in bed and non-pharmacological pain control^(28,29)^.

Realistic simulation is an active teaching methodology where theory and practice can be integrated in a safe and controlled environment. Participants have the opportunity to actively engage with the scenario, develop competencies, critical thinking, technical and non-technical skills, communication, and teamwork. For the success of a realistic simulation scenario, detailed planning is essential. Clear learning objectives should be defined to provide a high-quality experience, allowing participants to make decisions and solve problems found. The scenario planning process requires time, as each step must be validated, implemented, and tested. The use of these instruments aims to organize and systematize the process to achieve the defined objectives. In the context of this study, scenario construction followed the International Nursing Association for Clinical Simulation and Learning recommendations^(10)^. Additionally, evidence related to pain management by the nursing team in the PACU was gathered through a literature review conducted by the research team. The proposed scenario objectives were based on Bloom’s Taxonomy^(14,15)^, incorporating psychomotor, affective, and cognitive skills, all addressed together. The target group’s level of instruction and knowledge were considered to align with the scenario objectives^(15)^. The proposed high-fidelity simulation scenario included the phases of pre-briefing, briefing, simulation, and debriefing^(10,16,17)^.

Validity is a stage in scenario development aimed at ensuring that objectives and expected outcomes are achieved^(10)^. Despite recommendations emphasizing the importance of scenario validity, most high-fidelity simulation studies still do not undertake this step. Au *et al*.^(11)^ found that 81.8% of the studies in their sample did not validate their scenarios. This study demonstrated that validated high-fidelity scenarios, when compared to non-validated ones, significantly impact nursing students’ knowledge acquisition. In the case of our study, adjustments were necessary in the debriefing both in the validity and testing phases. This reinforces the importance of this step in ensuring that scenarios achieve the expected impact on nursing student learning and contribute to the consistency of the simulation-based experiences for learners^(10)^. Also, the scenario test is essential to ensure the scenario’s conceptual fidelity. It allows for an analysis of whether the scenario elements relate realistically, particularly if patient and case make sense to students^(10)^. This highlights the importance of ensuring that the validity of realistic simulation scenarios is carried out with the utmost scientific rigor while ensuring that its objectives are achieved.

Although significant advances have been made in recent years in recognizing the importance of scenario validity, there are challenges that need to be overcome. For instance, there is no consensus or even recommendations on which validated instruments for scenario construction and validity could be used. Therefore, further studies that contribute to advances in this topic to better align evidence-based recommendations are needed. Furthermore, there are differences between scenario validity studies, such as scenario area and objectives, the level of fidelity, the type of instrument used to construct and validate the scenario and whether it is validated or not, the number of experts, the number of rounds to reach the CVI, the way the data is analyzed, and the way the scenario is tested. Despite this, the studies have proved to be robust, achieving adequate validity rates.

The validity of high-fidelity scenarios has been a concern in different areas of nursing, including mental health and family care^(30)^, sepsis^(31,32)^, opioid use disorder^(33)^, and pediatric oncology^(34)^. There are different ways of assessing the proposed scenarios, with experts unanimously analyzing them^(30-32,34)^, but also including family members affected by the health problem^(33)^. The most commonly used form of data presentation and analysis is CVI^(30-32,34)^, but it is possible to successfully carry out focus group interview analysis^(33)^. Validity studies have found adequate CVI values ranging from 0.8^(30)^ to 1.0^(32)^. A study proposing a sepsis scenario found CVI > 0.9 in an analysis carried out by nine experts^(31)^. Another study proposing a simulation scenario in pediatric oncology family-focused care found a CVI of 0.92 in an analysis carried out by 35 experts^(34)^. This data shows that there are variations in the number of experts adopted in studies. Its main impact can be seen in the CVI, and it is considered ideal to include three to five to achieve a CVI of 0.78 and six to ten to find values higher than 0.9^(19)^. Despite this recommendation, it is important that researchers analyze the needs of each study in order to make the necessary adjustments. Accordingly, the present study proposed the number of experts in line with recommendations in literature, and obtained robust data to ensure that the proposed scenario can be considered validated.

Previous studies recommend the adoption of scenario-based simulation in nursing undergraduate curricula^(9)^, and emphasize the importance of scenario validity^(6)^ to enhance students’ learning. Furthermore, disseminating the methodological study in a way that allows scenarios to be carried out in other contexts can contribute to the practical application of theoretical knowledge related to pain management and other areas involving nursing care^(5-9)^. This positively impacts pain assessment and control by nursing teams, ultimately improving the quality of services provided. In addition, reproducing the scenario in different contexts^(17)^ and at different levels of student experience can improve and reinforce methods. The proposed and validated scenario in this study is comprehensive and aligns with international recommendations for pain management by nursing teams, making it adaptable in various contexts.

### Study limitations

It is considered a limitation that no validated instrument was used for constructing the scenario or for validity by experts. This may imply a commitment to good simulation practices in scenario planning, elaboration, and execution. To mitigate this impact, it is suggested that future studies use validated instruments for constructing and validating scenarios. Another limitation was the loss of experts during the development of the study.

### Contributions to nursing, health, or public policy

This study contributes with the construction, validity, and testing of a scenario that allows a simulated experience of an important situation that impacts perioperative nursing care indicators, including patient satisfaction. Considering practical hospital experiences, there is no guarantee that undergraduate students can experience this scenario before graduating. Thus, this tool is expected to contribute to improvements in undergraduate courses learning. Moreover, the scenario can be developed aiming to promote education and training for nurses working in the PACU.

## CONCLUSIONS

The proposed and validated scenario in this study is comprehensive and aligns with international recommendations for pain management by nursing teams, making it adaptable in various contexts.

## Data Availability

Data available upon request.
